# Synthesis of organophosphorus (III) compounds from white phosphorus enabled by a tandem electrothermal process

**DOI:** 10.1093/nsr/nwaf117

**Published:** 2025-03-27

**Authors:** Kaiyao Feng, Hua Zhang

**Affiliations:** School of Chemistry and Materials Science, South-Central Minzu University, China; School of Chemistry and Materials Science, South-Central Minzu University, China

Trivalent organophosphorus compounds [OPCs (III)] serve as essential functional molecules with broad applications including antioxidants, agrochemicals and catalytic ligands [1]. However, the industrial synthesis of them remains constrained by a reliance on phosphorus trichloride (PCl_3_) that is derived from white phosphorus (P_4_) through hazardous chlorination processes. Recent advancements in electrochemical synthesis have highlighted its potential as a sustainable alternative due to inherent atom economy and reduced environmental impact [2–4]. Although the direct electrooxidation of P_4_ to OPCs (III) could circumvent traditional environmental concerns, there is a critical thermodynamic barrier to practical implementation: the lower oxidation potential of OPCs (III) relative to P_4_ inevitably causes overoxidation during electrolysis.

**Figure 1.**
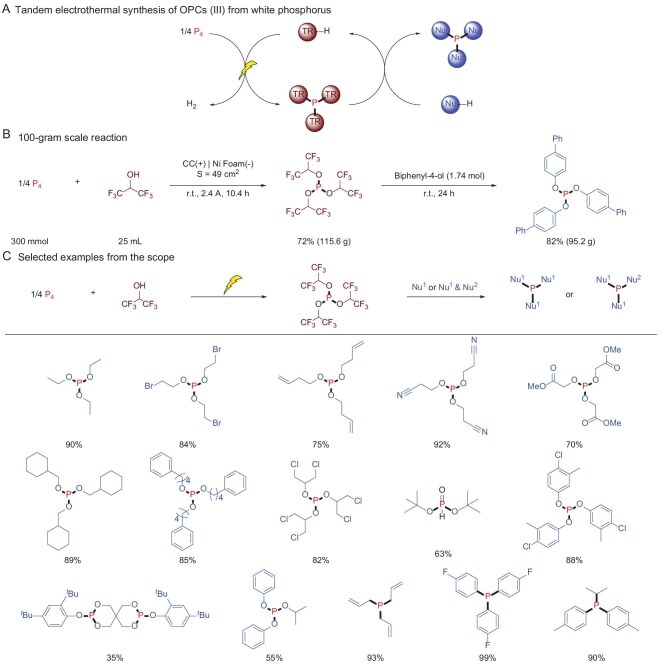
Tandem electrothermal synthesis of OPCs (III) from white phosphorus.

To address this challenge, Lei *et al.* recently demonstrated a tandem electrothermal strategy that overcomes the inherent redox incompatibility [5]. The process begins with the electrochemical oxidation of P_4_ in the presence of a recyclable electron-deficient transfer reagent (TR-H), which generates an electrophilic intermediate P(TR)_3_ (Fig. 1A). The intermediate exhibits enhanced oxidation resistance compared with P_4_ and functions as a versatile phosphorus transfer agent that is analogous to PCl_3_. Subsequent thermochemical conversion yields diverse trivalent phosphorus compounds while regenerating TR-H, establishing a closed-loop system. Hexafluoroisopropanol (HFIP) was optimized as the TR-H, enabling the efficient synthesis of P[OCH(CF_3_)_2_]_3_. Electrostatic potential surface calculations indicate that –CF₃ groups convert the phosphorus center from nucleophilic to electrophilic by dispersing electron density via the strong electric field of highly electronegative fluorine atoms, thus enhancing oxidation resistance and phosphorus transfer efficiency.

The reaction efficiency was further enhanced by a tetrabutylammonium iodide (TBAI)-4-dimethylaminopyridine (DMAP) catalytic adduct. Mechanistic investigations showed that anodically generated iodine species activate P_4_ to form P–I intermediates, which subsequently react with a HFIP–DMAP hydrogen-bond complex to yield P–O bonds in the final product. This dual catalytic system not only accelerates P_4_ electrooxidation, but also amplifies the nucleophilicity of HFIP. The methodology achieved remarkable scalability, producing 115.6 g of P[OCH(CF_3_)_2_]_3_ and 95.2 g of tri([1,1′-biphenyl]-4-yl) phosphite in a single batch (Fig. 1B). Notably, the process accommodates fluctuating renewable energy (wind and solar power) inputs while maintaining operational stability and the P[OCH(CF_3_)_2_]_3_ reagent demonstrates broad substrate compatibility, successfully converting diverse nucleophiles into various OPCs (III) such as trialkyl and triaryl phosphites, phosphines and related derivatives (Fig. 1C).

This work establishes a paradigm for sustainable OPC (III) synthesis by resolving the long-standing overoxidation problem in P_4_ electrochemistry. The tandem electrothermal approach not only enables scalable production, but also aligns with green-chemistry principles through energy-efficient operation and renewable-energy integration, marking a critical step toward environmentally benign organophosphorus production.
